# Mapping Meiotic Single-Strand DNA Reveals a New Landscape of DNA Double-Strand Breaks in Saccharomyces cerevisiae

**DOI:** 10.1371/journal.pbio.0050324

**Published:** 2007-12-11

**Authors:** Cyril Buhler, Valérie Borde, Michael Lichten

**Affiliations:** Laboratory of Biochemistry and Molecular Biology, National Cancer Institute, National Institutes of Health, Bethesda, Maryland, United States of America; Brandeis University, United States of America

## Abstract

DNA double-strand breaks (DSBs), which are formed by the Spo11 protein, initiate meiotic recombination. Previous DSB-mapping studies have used *rad50S* or *sae2Δ* mutants, which are defective in break processing, to accumulate Spo11-linked DSBs, and report large (≥ 50 kb) “DSB-hot” regions that are separated by “DSB-cold” domains of similar size. Substantial recombination occurs in some DSB-cold regions, suggesting that DSB patterns are not normal in *rad50S* or *sae2Δ* mutants. We therefore developed a novel method to map genome-wide, single-strand DNA (ssDNA)–associated DSBs that accumulate in processing-capable, repair-defective *dmc1*Δ and *dmc1*Δ *rad51*Δ mutants. DSBs were observed at known hot spots, but also in most previously identified “DSB-cold” regions, including near centromeres and telomeres. Although approximately 40% of the genome is DSB-cold in *rad50S* mutants, analysis of meiotic ssDNA from *dmc1Δ* shows that most of these regions have substantial DSB activity. Southern blot assays of DSBs in selected regions in *dmc1Δ*, *rad50S*, and wild-type cells confirm these findings. Thus, DSBs are distributed much more uniformly than was previously believed. Comparisons of DSB signals in *dmc1*, *dmc1 rad51*, and *dmc1 spo11* mutant strains identify Dmc1 as a critical strand-exchange activity genome-wide, and confirm previous conclusions that Spo11-induced lesions initiate all meiotic recombination.

## Author Summary

During meiosis, the two copies of each chromosome present in the full (diploid) genome come together and then separate, forming haploid gametes (sperm and eggs, in animals). Recombination, which swaps DNA between chromosomes, is critical for chromosome pairing and separation, and also promotes genetic diversity in the next generation, providing the feedstock for evolution. DNA double-strand breaks (DSBs), which are formed by the conserved Spo11 nuclease, initiate meiotic recombination. DSB mapping is thus an alternative to standard genetic analysis for determining where meiotic recombination occurs. DSBs have been most extensively mapped in budding yeast mutants that fail to remove Spo11 from break ends, blocking further recombination steps. Paradoxically, those studies indicated that DSBs are absent from large regions where recombination was known to occur. We developed a new DSB mapping method that purifies and analyzes the single-strand DNA formed at breaks after Spo11 removal. This new map shows that DSBs (and by inference, recombination) actually occur frequently throughout almost all of the budding yeast genome, in a distribution that is consistent with recombination's roles in chromosome pairing and in generating genetic diversity. This new mapping method will be useful for studying meiotic recombination and DNA damage repair in other organisms.

## Abstract 3

The authors developed a new method to detect DNA damage genome-wide, and they used it to show that meiotic recombination is more uniformly distributed in budding yeast than was previously believed.

## Introduction

Meiosis results in the faithful and efficient division of a diploid genome into four haploid gametes. After one round of DNA replication, cells undergo two rounds of chromosome segregation. Recombination between homologous chromosomes (homologs) occurs during prophase of the first division. Meiotic recombination promotes genetic diversity, but its main role is to ensure interhomolog association during the first meiotic division [1]. This association is absolutely required for efficient homolog separation, and defects in meiotic recombination result in chromosome nondisjunction [2].

Meiotic recombination is initiated by DNA double-strand breaks (DSBs) [3]. DSBs are formed by Spo11, a homolog of the catalytic subunit of a type II DNA topoisomerase [4,5]. Spo11 is conserved among eukaryotes, and loss-of-function Spo11 mutants have been shown to be meiotic recombination–defective in many organisms [6–11]. DSBs form by a mechanism that involves the covalent attachment of Spo11 to break ends [5,12]. Subsequent to DSB formation, Spo11 is removed by endonucleolytic cleavage [13], and break ends undergo 5′ to 3′ resection to create 3′ end single-strand tails [14]. This produces a substrate for Dmc1 and Rad51, which are eukaryotic RecA homologues that catalyze the strand-invasion step of meiotic DSB repair by interhomolog recombination [15,16]. Dmc1 is expressed only during meiosis and is responsible for the bulk of meiotic DSB repair, whereas Rad51 is required for homologous recombination during vegetative growth and also contributes to meiotic recombination [17,18].

Meiotic DSBs form in early meiosis I prophase, after premeiotic S phase [19]. DSB formation appears to be co-regulated with DNA replication in two ways. Replication and DSB formation both require active cyclin-dependent kinase (Cdc28) and the B-type cyclin Clb5 [20–23]. DNA replication and DSB formation also are temporally linked at the chromosome level, in that delaying replication of the left arm of chromosome *III* (chr *III-L*) causes a similar delay in DSB formation specifically on that chromosome arm [19]. Although it remains to be determined how DSB formation can be temporally linked to replication, it also appears that DSBs can form in the absence of DNA replication, because mutants lacking the replication licensing factor Cdc6 form meiotic DSBs in the absence of bulk DNA replication [24]. Hochwagen and Amon have proposed that replication initiation activates a checkpoint system that prevents DSB formation in unreplicated DNA [25].

Certain point mutations in *RAD50* (*rad50S*) and in *MRE11* (*mre11–58* and *mre11S*), as well as deletions of the *SAE2/COM1* gene, which encodes a protein that appears to regulate activity of the Mre11/Rad50/Xrs2 complex, have been widely used in characterizing early steps in DSB formation and in determining DSB distributions [3,26–31] . In these mutants, hereafter referred to as *rad50S-*like, DSBs accumulate unprocessed and unrepaired, with Spo11 covalently attached [5], which has facilitated the chromosome- and genome-wide DSB mapping ([32–34] and reviewed in [31,35]). In these *rad50S*-like mutants, DSB hot spots are distributed unevenly. Chromosomes are partitioned into large (≥50 kb) domains with many DSB hot spots, alternating with domains of similar size where DSBs are reduced or absent, even though potential DSB sites (i.e., chromatin nuclease hypersensitive sites [36,37]) are present [38]. These “DSB-cold” regions are generally found at chromosome ends and adjacent to centromeres, but occur at many other locations as well [33,34]. *Schizosaccharomyces pombe rad50S* mutants also show a nonuniform DSB map, with most breaks occurring at sites in tight (<3 kb) clusters separated by ∼50 kb DSB-cold regions [39,40].

Two independent observations suggest that studies using *rad50S*-like mutants have underestimated DSB levels in S. cerevisiae. First, although DSBs rarely occur in a 30-kb centromere-proximal region of chromosome *III*, both the standard genetic map [41] and genetic studies [38] indicate that crossovers frequently occur in this region. Second, delaying DSB formation by 1 h on chr *III-L* causes a 4- to 5-fold reduction in DSB levels on that chromosome arm in *rad50S*-like mutants, but not in wild-type cells [19]. These findings suggest that DSB maps from *rad50S*-like mutants underestimate true DSB frequencies in some parts of the genome.

To test this suggestion, we analyzed DSB distributions in mutants lacking Dmc1 or both Rad51 and Dmc1 strand-exchange proteins. In these mutants, Spo11 is removed from break ends, and unrepaired DSBs accumulate with 3′-ended ssDNA tails [17]. We reasoned that purification of this ssDNA could be used to enrich break-adjacent sequences, and thus to map DSBs at a whole-genome level. Using this new mapping strategy, we obtained a whole-genome distribution of meiotic DSBs that is considerably more uniform than was previously described, with substantial DSB levels in regions previously thought to be DSB-free. Our whole-genome DSB analysis also confirms that Spo11 forms all the lesions that initiate meiotic recombination, and that Dmc1 is a critical meiotic recombinase in all regions of the genome.

## Results

### DSB Frequencies in *dmc1*Δ Are Independent of DSB Timing

**Figure 1:**
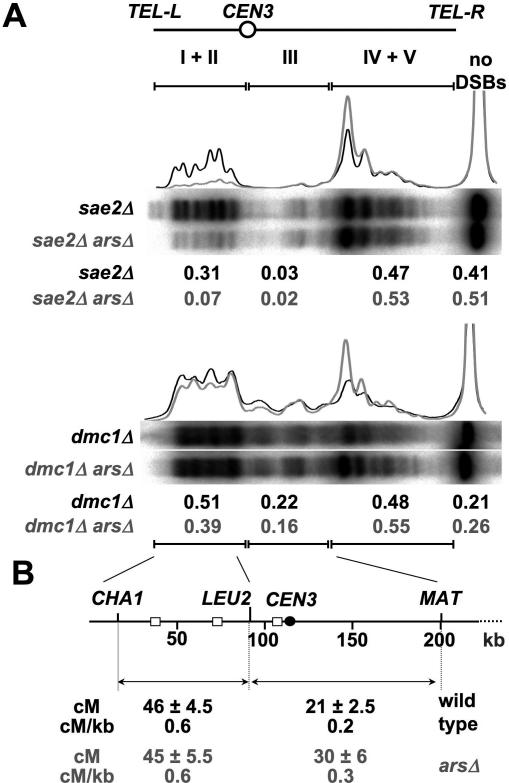
Delaying Replication Does Not Affect Crossing-Over in Wild Type or DSB Frequencies in *dmc1*Δ (A) Southern blots of pulsed-field gels, probed with a left end–adjacent probe (nt 15,838–16,857) to detect DSBs on chromosome *III* in a normal chromosome or one with all three active replications origins on chr *III-L* deleted (*arsΔ*). DNA was prepared from *sae2*Δ (MJL2306), *sae2*Δ *ars*Δ (MJL2529), *dmc1*Δ (MJL2560), and *dmc1*Δ *ars*Δ (MJL2683) 6 h after initiation of meiosis. Quantification traces for each lane, normalized to total lane intensity, are shown (normal *chr III-L*, black; *arsΔ*, gray). DSB frequencies in the left arm (I + II), central “cold” region (III), and right arm (IV + V) domains [32] and frequencies of uncut chromosomes are expressed as fraction of total lane signal. Values are corrected to account for double cutting events (see Materials and Methods). The uncorrected values are as follows: for interval I + II: *sae2Δ*, 0.27; *sae2Δ arsΔ*, 0.07; *dmc1Δ*, 0.42; *dmc1Δ arsΔ*, 0.33. For interval III: *sae2Δ*, 0.02; *sae2Δ arsΔ*, 0.02; *dmc1Δ*, 0.13; *dmc1Δ arsΔ*, 0.11. For interval IV + V: *sae2Δ*, 0.3; *sae2Δ arsΔ*, 0.4; *dmc1Δ*, 0.24; *dmc1Δ arsΔ*, 0.3. (B) Frequencies of crossing-over in the indicated intervals (centiMorgans ± standard error), in wild type (MJL3237) or strains with an *arsΔ* chr*III-L* (MJL3236). Black circle indicates centromere; white squares indicate replication origins (*ARS305, ARS306, ARS307*) that are deleted in replication-delayed chromosome arm.

Borde et al. showed that deleting all active replication origins from chr *III-L* caused a delay in DSB formation in wild-type cells but did not alter DSB levels. By contrast, the same origin-deleted chr *III-L* showed a 4- to 5-fold reduction in DSBs in the *rad50S*-like mutant *sae2Δ*, which is unable to remove Spo11 from DSB ends [19]. We extended this analysis to *dmc1Δ* mutants, where DSBs accumulate at a stage after Spo11 is removed from break ends. Southern blots of pulsed-field gels were used to detect DSBs along the entire chromosome (Figure 1A). In agreement with previous data, late DSB formation on chr *III-L* was associated with a 4-fold reduction in DSBs in *sae2Δ* cells. In contrast, *dmc1*Δ mutants showed similar DSB frequencies on normal and DSB-delayed chromosome arms. This observation suggests that DSB levels measured in *dmc1Δ* mutants might better represent recombination activity in wild type. Consistent with this suggestion, wild-type cells showed similar frequencies of crossing-over in wild type and DSB-delayed chr *III-L* (Figure 1B). Meiotic intragenic recombination on chr *III-L* has also been shown to be independent of DSB timing [19].

Comparison of DSB patterns on normal chromosomes *III* in *sae2Δ* and *dmc1Δ* strains (Figure 1A) revealed two other differences. First, the fraction of chromosomes that suffer DSBs was greater in *dmc1*Δ (75%–80%) than in *sae2Δ* (50%–60%), and the total DSB frequency (1.2 DSBs/chromosome) in *dmc1Δ* was substantially greater than in *sae2Δ* (0.8 DSBs/chromosome). Second, DSBs formed in the center of the chromosome (region III) in about 20% of chromosomes in *dmc1Δ* cells, but in almost none in *sae2Δ* (Figure 1A, previously reported by Blat et al. [42] and by Dresser et al. [43]). The substantial DSB signal seen in region III in *dmc1Δ* mutants is sufficient to account for the meiotic recombination observed in this region in wild-type cells (Figure 1B), in contrast to the very low frequency of DSBs seen in this region in *rad50S*-like mutants ([32,34,42], Figure 1A).

### DSB-Specific ssDNA Enrichment with Benzoyl Naphthoyl DEAE Cellulose

**Figure 2:**
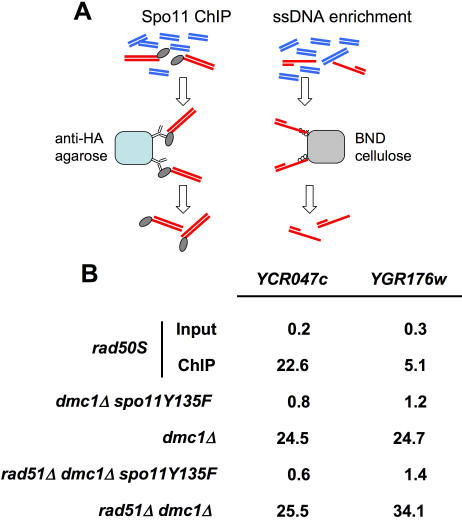
Enrichment of DSB-associated DNA (A) Cartoon illustrating enrichment procedures used (see Protocol S1 for details). DSB ends formed in *rad50S* can be enriched by immunoprecipitation of Spo11 covalently linked to break ends (here with antibody directed against a C-terminal 3xHA tag). DSBs formed in *dmc1Δ* and *rad51Δ dmc1Δ* can be enriched by BND cellulose purification of ssDNA ends. (B) Quantitative PCR measurement of enrichment relative to ribosomal DNA (rDNA) for sequences near DSB hot spots (*YCR047c* and *YGR176w*). Spo11 enrichment ratios for *rad50S* (MJL1083) were determined from input samples and immunoprecipitates (ChIP). ssDNA enrichment ratios for *dmc1*Δ (MJL3095), *spo11Y135F dmc1*Δ (MJL3096), *rad51*Δ *dmc1*Δ (MJL3272), and *spo11Y135F rad51*Δ *dmc1*Δ (MJL3274) were determined using BND cellulose eluates. DSB frequencies at *YGR176w* and *YCR047c*, as determined on Southern blots, are ∼10% of chromosomes (Figure 5 and unpublished data); thus, the overall selectivity for DSBs in both Spo11 ChIP and ssDNA enrichment is 200–300-fold above background.

The above data indicate that more DSBs are formed in *dmc1Δ* mutants than in *rad50S-*like mutants, and that DSBs are artificially low in some regions in *rad50S-*like mutants. We therefore developed a strategy to measure DSB levels in recombinase-deficient strains, taking advantage of the ssDNA that accumulates on each side of DSBs in *dmc1*Δ mutants [17] and in *rad51*Δ *dmc1*Δ mutants [44,45]. Benzoyl naphthoyl DEAE (BND) cellulose, which selectively binds ssDNA [46–48], was used to enrich for these DSB-associated sequences (see Materials and Methods), which were compared to DSB-associated sequences prepared from *rad50S* mutants by immunoprecipitation of Spo11-linked DNA [33,34]. Quantitative PCR analysis of DNA prepared by both methods (Figure 2) showed similar enrichment at two DSB hot spots (*YCR047c* and *YGR176w* [32,34,49]) relative to ribosomal RNA genes, where meiotic DSBs are absent [50] and meiotic recombination is infrequent [51,52]. No ssDNA enrichment was seen in the DSB-negative *spo11-Y135F dmc1Δ* and *spo11Y135F rad51Δ dmc1Δ* mutants, indicating that all meiotic ssDNA at these sites originates from Spo11-induced DSBs.

### Whole-Genome Mapping of ssDNA Reveals New Meiotic DSB Sites

We mapped break-associated DNA sequences with oligonucleotide-based microarrays [53] that offer greater resolution (average interelement distance of 290 nucleotides [nt]) as well as more uniform element-to-element hybridization properties than do the spotted PCR-product microarrays used previously [33,34]. To allow direct comparison between different array datasets, we developed a background-based normalization procedure, rather than the more commonly used median normalization, because the latter method results in an artifactual lowering of array signals when a positively skewed experimental signal (typical of signals from chromatin immunoprecipitation [ChIP]-chip experiments) is compared to a more symmetrically distributed background signal [54]. Enrichment values (Table S1) were background-normalized using the median signal from a set of probes located >2 kb from either end of the coding sequences of 19 large genes (see Materials and Methods, Table S2). Because the vast majority of DSBs occur in promoter regions [32,37,55], these probes are unlikely to be present in either meiotic ssDNA or in Spo11-associated DNA.

**Figure 3:**
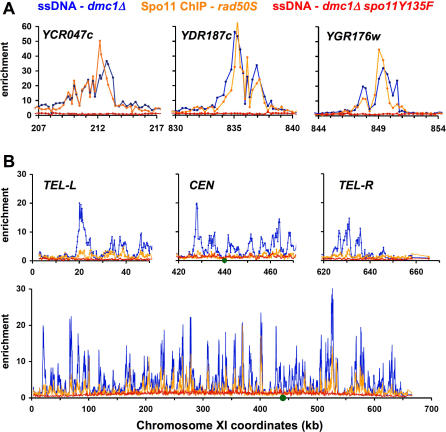
Examples of Concordance and Discordance between DSBs in *dmc1*Δ and *rad50S* In each plot, normalized, unsmoothed array signals (average of two experiments) are as follows: orange, Spo11 ChIP from *rad50S* (MJL1083); blue, BND cellulose–enriched ssDNA from *dmc1*Δ (MJL3095); red, BND cellulose–enriched ssDNA from *spo11Y135F dmc1*Δ (MJL 3096). (A) Three concordant regions where DSB signals are similar in *rad50S* and *dmc1Δ*. *x*-axes, chromosome coordinates (kb) on chromosomes *III* (*YCR047c*), *IV* (*YDR187c*), and *VII* (*YGR176w*). (B) DSB signals on chromosome *XI*. Insets show three discordant regions where the DSB signal in *dmc1Δ* is much greater than in *rad50S*. Green dot indicates the centromere.

Background normalization resulted in datasets with very similar dynamic ranges, irrespective of the DSB enrichment method used, either ssDNA from *dmc1Δ* or Spo11 ChIP material from *rad50S* (Figure 3, Tables S1 and S7, and Figures S1–S3). Similar DSB signals were seen in both *dmc1Δ* and in *rad50S* at three of the strongest previously identified DSB hot spots (Figure 3A) [32,34], and all of the top 50 DSB hot spots in *rad50S* were also present among the *dmc1Δ* hot spots (Table S3).

The current oligonucleotide (oligo)-array analysis of Spo11-linked DNA from *rad50S* is in good agreement with previous analyses using PCR-product arrays [33,34]. Whereas Borde et al. identified 585 DSB hot spots in *sae2*Δ mutants [34], the increased dynamic range and resolution of oligo-arrays allowed identification of 1,306 DSB hot spots (peak values of 2–30 times background) in *rad50S* samples, and these included most of the previously identified hot spots (Tables S4 and S5).

To ask if all ssDNA detected in a *dmc1Δ* mutant was DSB-associated, we first examined the Spo11-dependence of the meiotic ssDNA signal. No enrichment of ssDNA above background was observed in any region in a *spo11Y135F dmc1*Δ mutant (Figure 3, Table S1, and Figure S3), consistent with Spo11-catalyzed DSBs being the primary lesion-initiating meiotic recombination genome-wide. We also asked if all DSBs repairable by homologous recombination are detected in *dmc1Δ* mutants. Dmc1 and Rad51 both catalyze strand exchange during meiosis, and Dmc1 has been shown to be responsible for the bulk of meiotic DSB repair at a few defined DSB sites [17,18]. We asked if any regions of the genome contained substantially more ssDNA in *rad51Δ dmc1Δ* than in *dmc1Δ* strains, as might be expected if the majority of DSBs in some regions were repaired in a Rad51-dependent, Dmc1-independent manner. Similar DSB distributions were seen in the two mutant backgrounds (Tables S1 and S7, Figure S2), and no DSB peaks were 2-fold greater in *rad51Δ dmc1Δ* than in *dmc1Δ* (Figure S2, Table S1, and unpublished data). In addition, substantially lower ssDNA and DSB levels were detected in meiotic DNA sample taken at the same time in meiosis from a *rad51Δ* single mutant (Tables S1 and S7, Figure S4, and unpublished data). These results indicate that Dmc1 is critical for the majority of meiotic DSB repair reactions in all regions of the genome. For this reason, further ssDNA distribution analysis will use data from *dmc1Δ* single mutants.

### Meiotic DSBs Are More Frequent and More Uniformly Distributed in *dmc1*Δ than in *rad50S*

Although some DSB hot spots display similar enrichment in *dmc1Δ*- and *rad50S*-derived material, disparity between the two mutants is seen in many parts of the genome (Figure 3 and Figure S1). The majority of nonbackground array elements display greater enrichment in *dmc1Δ* than in *rad50S* (Figure S5 and Table S7); in 40% of array elements, the *dmc1Δ*/*rad50S* signal ratio was greater than 2 (Figure S5). In addition, DSB-cold regions were notably absent from *dmc1Δ* mutants. We identified 260 DSB-cold regions longer than 10 kb (all elements <2× background) in *rad50S*, representing about 4.8 Mb, or 40% of the single-copy genome. Only 28 of these regions (about 370 kb, or 3% of the single-copy genome) were also DSB-cold in *dmc1Δ* mutants (Table S1 and Figure S5). The majority of the >10-kb regions were at loci expected to be DSB-cold: 14 were near chromosome ends; eight contained very large open reading frames, and one contained a centromere. Thus, a substantially greater fraction of the genome is DSB-associated in *dmc1Δ* than in *rad50S* mutants, and only a very small fraction of regions can be described as DSB-cold.

**Figure 4:**
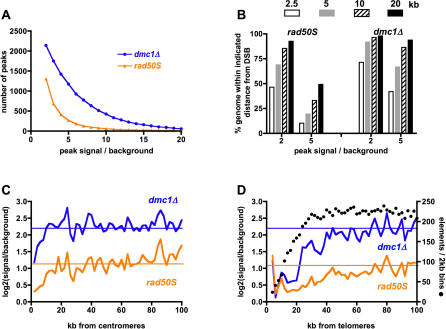
Summary of DSB Distributions (A) The number of DSB hot spots in *dmc1Δ* (blue) exceeds the number in *rad50S* (orange) at all DSB intensities. DSB peaks were identified from smoothed data (Materials and Methods). Total DSB hot spots with peak heights greater than the indicated multiple of background are plotted. (B) More of the genome is close to a DSB in *dmc1Δ* than in *rad50S*. The graph shows the fraction of the genome less than the indicated distance from a DSB peak with a height greater than 2-fold or 5-fold above background. (C) DSBs are reduced in a 8–10-kb region near centromeres. All 32 chromosome arms were aligned at the centromere, and the average unsmoothed enrichment signal was determined for all elements in 2-kb bins (>200 array elements/bin). Horizontal lines indicate genome-wide average. (D) DSB activity near chromosome ends. All 32 chromosome arms were aligned at chromosome ends, and the average enrichment signal was determined in 2-kb bins as for centromeres. Horizontal lines indicate genome-wide average. Black dots indicate number of array elements per 2-kb bin with homology to the SK1 strains used here (see Materials and Methods).

**Figure 5:**
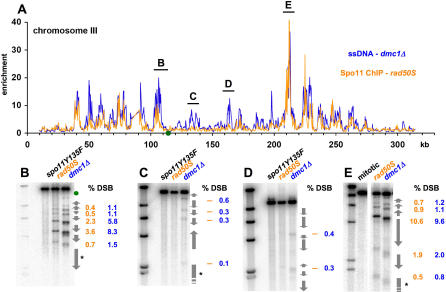
Confirmation of DSB Patterns (A) Spo11 ChIP ratios from *rad50S* (orange) and BND cellulose enriched ssDNA ratios from *dmc1*Δ (blue) on chromosome *III*. Green dot indicates the centromere. (B–E) Southern blot detection of DSBs in the indicated regions. Blots contain DNA from meiotic (5 h) samples of *spo11-Y135F dmc1*Δ (MJL3096), *dmc1*Δ (MJL3095), and *rad50S* (MJL1083) cells, and DNA from mitotic wild-type (MJL1578) cells. DSB frequencies (% total lane signal) are indicated to the right of each blot (- denotes no signal detected above background). Blots were hybridized with radioactive probes (*) internal to *YCL011c*, *YCR007c*, *YCR052w* open reading frames (for (B), (C), and (E), respectively; details supplied upon request), and *YCR019w* in (D). Restriction enzymes used: (B and C): *Xho*I; (D): *Pvu*II; and (E): *Bgl*II. DNA length standards (first lane in each blot) contain a *Hin*dIII digest of phage λ DNA.

The discordance between *dmc1Δ* and *rad50S* is further illustrated by an examination of the number of DSB hot spots/genome and inter–hot spot distances at different peak intensity thresholds (Figure 4A and Table S5). At all peak intensities, the number of DSB hot spots in *dmc1*Δ exceeded the number in *rad50S*. When the strongest hot spots are considered (peak/background > 5), approximately five times more hot spots are present in *dmc1*Δ than in *rad50S* (Figure 4A). At a lower threshold (peak/background > 2), about twice as many hot spots are present in *dmc1*Δ as are in *rad50S*. This convergence is consistent with the suggestion that DSBs are formed at the same sites in both mutant backgrounds, but the DSB intensity in *rad50S* is substantially less than in *dmc1*Δ at many sites ([19], see also Figures 1 and 5).

As expected from the greater DSB hot spot density, inter–hot spot distances in *dmc1*Δ are substantially less than in *rad50S* (Table S5). At twice the background threshold, the mean inter–hot spot distance is about 5.5 kb in *dmc1Δ* but about 8.5 kb in *rad50S*. This discordance is even greater with stronger DSB hot spots (≥5 × background), with a mean interpeak distance of 9.5 kb for *dmc1Δ* and about 35 kb for *rad50S*. This discordance is reflected in differences in calculated fractions of the genome within a given distance of the nearest DSB (Figure 4B). In *dmc1Δ*, more than 70% of the single-copy yeast genome is less than 2.5 kb from a DSB peak that is twice background, as compared to less than half of the genome in *rad50S*. Taken together, these observations clearly indicate that DSBs occur more frequently, and are more uniformly distributed, in *dmc1*Δ than in *rad50S*.

### DSBs Form near Centromeres and Chromosome Ends

Studies of *rad50S-*like mutants indicate that DSBs are absent from sequences within 20 kb of centromeres and within 40–50 kb of chromosome ends [33,34]. The increased DSB density in *dmc1Δ* prompted a re-examination of DSB signals near centromeres and chromosome ends (Figure 4C and 4D). In contrast to the 20-kb centromere-adjacent DSB-cold regions seen in previous studies, we observed significantly reduced DSB signals only in an 8-kb or 10-kb window for *dmc1Δ* and *rad50S*, respectively (Figure 4C). However, individual chromosomes display DSB peaks within this centromere-proximal zone (Figure 3, Figure S1, and unpublished data) [56], and average *dmc1Δ* element signals in the 2 kb immediately centromere-proximal are significantly greater than background (*p* < 0.001, Mann-Whitney test). Taken together, these data suggest that DSBs are absent from centromeres themselves, with the likelihood of DSB formation rising with distance over the next 8–10 kb.

DSB signals are also significantly reduced, relative to the genome-wide average, in a ∼60-kb region at chromosome ends (as defined by the S. cerevisiae reference sequence). Average DSB signals in the ∼20 kb closest to chromosome ends are close to background. In *dmc1Δ*, the DSB signal is ∼2/3 of the genome-wide average in the next 20 kb. In the region 40–60 kb from chromosome ends, DSBs in both *dmc1Δ* and *rad50S* approach, but are still significantly below, the genome-wide average (80%–90%, *p* <0.0001, Mann-Whitney test). These data indicate that DSBs are absent from the 20 kb closest to most chromosome ends, and are present at modestly reduced frequencies in the next 20 kb. These conclusions must be conditioned by the fact that uncertainty exists regarding the precise distance of many array elements from chromosome ends in our study. In particular, sequences near some chromosome ends are known to differ between the strain in which this study was performed (SK1) and S288c, the reference sequence strain used in microarray design ([32], E. J. Louis, personal communication).

### Validation of Microarray-Based DSB Maps

Southern blot analysis of DSBs in selected regions confirmed the conclusion, from whole-genome data, that regions exist where DSB levels in *dmc1Δ* are substantially greater than in *rad50S*. Similar DSB frequencies were measured at the *YCR047c* hot spot in *dmc1*Δ and *rad50S*, both on arrays (Figures 3A and 5A) and on Southern blots (Figure 5E)*,* although DSB fragments differed in size, due to the 5′-to-3′ single-strand resection that occurs in *dmc1Δ* but not in *rad50S*. Agreement between array and Southern-based DSB frequencies in *dmc1Δ* and *rad50S* was also observed at a second DSB hot spot (*YDR187c*; Figure S6). Southern blot analysis at several loci also confirmed the regional discordance between *dmc1Δ* and *rad50S* microarray data. In the central region of chromosome *III*, significant DSB levels were detected near the promoter regions of *YCR011c*, *YCR020c*, and *YCR022c* in *dmc1Δ* but not in *rad50S*, in both microarrays (Figure 5A) and Southern blots (Figure 5C and 5D). A 3-fold difference between *dmc1Δ* and *rad50S* in DSBs in the region surrounding *YCL011c* seen in microarrays (Figure 5A) was also confirmed on Southern blots (Figure 5B). Similar validation was obtained from analysis of other discordant loci, including a 30-kb region at the end of chromosome *XIII* and at *YIR020c* (Figure S6).

**Figure 6:**
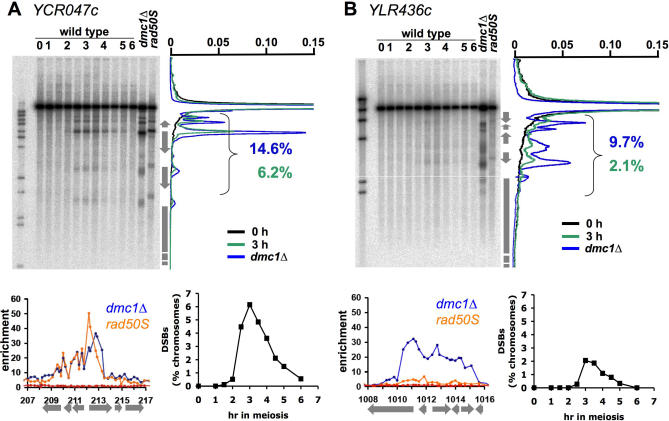
DSBs During a Wild-Type Sporulation (A) DSBs in the concordant *YCR047c* region. Upper left: Southern blot showing DSBs in DNA from wild type (MJL1578, indicated hr after initiating sporulation), *dmc1Δ* (MJL3095, 5 h), and *rad50S* (MJL1083, 5 hr). Arrows indicate open reading frames in the region, top to bottom; *YCR047c*; *YCR048w*; *YCR051w*; *YCR052w* (probe). Digest: *Bgl*II. DNA length standard (first lane) contains a *BstE*II digest of phage λ DNA Upper right: density trace, normalized to total lane density, of the indicated lanes. DSB peak intensities (% total lane density) are sum of all 5 detectable DSB bands. Lower left: background normalized average DSB signals from microarrays for the same region. Open reading frames, left to right: *YCR045c; YCR046c; YCR047c*; *YCR048w*; *YCR051w*; *YCR052w.* Lower right: DSB timing in wild type, for the DSB sum shown in density trace. (B) DSBs in the discordant *YLR436c* region. Panels as in (A). Open reading frames, top to bottom: *YLR440c*; *YLR439w*; *YLR438w*; *YLR437c*; *YLR436c* (probe). Digest: *BstE*II. DNA length standards (first lane) contain a *Hin*dIII digest of phage λ DNA.

The above Southern blot analysis confirms the microarray-based identification of regions of discordance between *rad50S* and *dmc1Δ* DSB maps. In one such region—the centromere-proximal region of chromosome *III*—genetic measures of recombination in wild type are more consistent with the DSB levels seen in *dmc1Δ* than in *rad50S-*like mutants (Figure 1; see also [38]). It was therefore of interest to determine whether breaks were frequent or infrequent in wild-type cells in regions of discordance between *rad50S-* and *dmc1-*based DSB map. We analyzed DSB levels and timing in wild-type cells in the *YCR047c* concordant regions and in three discordant DSB sites (*YOR347c*, *YLR436c*, and *YDL220c*), where breaks are present in *dmc1Δ* and almost absent in *rad50S* (Figure 6 and Figure S7). Substantial DSB levels were detected in wild type at all four loci. DSB peaks occurred at 3–3.5 h after initiation of sporulation at all loci; DSBs tended to occur at discordant loci later than at the concordant locus, although asynchrony in DSB formation and culture-to-culture variation precludes accurate temporal assignment (Figure 6, Figure S7, and unpublished data). These data confirm the conclusion that *dmc1Δ* mutants are better than *rad50S*-like in predicting whether or not DSBs occur at a given locus in wild type.

## Discussion

### A New Strategy to Map Recombination Initiation Events

All known homologous recombination mechanisms produce ssDNA, which is bound by RecA-like strand-exchange proteins and used to initiate recombination by invading a second duplex DNA molecule [15]. In mutants lacking RecA-like proteins, lesion-associated ssDNA is expected to accumulate. Approaches that detect this ssDNA should therefore detect the ensemble of recombination initiation events, irrespective of mechanism, as long as the ssDNA formed is stable. We have presented here the mapping and quantification of meiotic DSBs in S. cerevisiae, based on microarray analysis of break-associated ssDNA isolated by BND cellulose enrichment. A similar approach has been used by Blitzblau and coworkers, with similar conclusions [56]. This method has also been used to identify ssDNA regions that accumulate when mitotic replication is blocked [48]. Given the sensitivity and selectivity of this method (>200-fold, Figure 2), it should provide a powerful way to detect lesions that initiate homologous recombination, and should be generally applicable to many organisms, including mammals.

This method provides a powerful way to detect early recombination intermediates, but care must be taken when interpreting results. First, different ssDNA-containing intermediates may not be equally stable. For example, only recombination can repair ssDNA tracts with 3′ ends, the primary precursor in meiosis [14,55,57]. On the other hand, ssDNA with 5′ ends or ssDNA gaps can be filled by DNA polymerases. Such lesions might not persist as ssDNA, and thus would have been under-detected in our assay. Second, since 5′ to 3′ resection continues over time in *dmc1Δ* mutants [17,18], early-forming DSBs might be associated with more ssDNA than late-forming DSBs, thus giving a relatively stronger signal on arrays. However, since all DSBs we examined in *dmc1Δ* display a similar resection size (Figure 6, Figure S7, and unpublished data), we believe that a biased representation of early and late breaks is unlikely.

The whole-genome analysis of meiotic DSB distributions shows that some DSB hot spots show similar signals in *dmc1Δ* and in *rad50S*, but a greater number display a *dmc1Δ/rad50S* signal ratio of 2-fold or greater, and many DSB sites are detected in *dmc1Δ* but not in *rad50S* (Figure 4, Figures S1 and S5, and Table S1). This finding is confirmed by Southern blot analysis of DSBs in selected regions in *dmc1Δ* and *rad50S* (Figure 5 and Figure S6) with a linear relationship between DSB frequencies on Southern blot and on microarray (Figure S8). Break processing–capable but repair–defective mutants such as *dmc1Δ* are also better predictors of DSB locations in wild type than *rad50S*-like mutant (Figure 6 and Figure S7) and DSB frequencies in *dmc1Δ* agree with genetic distances measured at the chromosomal level (Figure 1). In addition, integrated whole-genome DSB signals from microarray analysis of *dmc1Δ* and *rad51Δ dmc1Δ* predict 140–170 DSBs/meiotic nucleus (Table S7), which approaches the genetic map-based estimate of 180–270 DSBs/nucleus [58]. This stands in contrast to the much lower estimate of about 44 DSBs/nucleus from *rad50S* data (Table S7). We conclude that the distribution of ssDNA-enrichment signals in DNA from *dmc1Δ* mutants is currently the most accurate representation of the relative distribution of DSBs in wild-type cells, although it is likely that it underestimates true DSB frequencies.

### A Revised Whole-Genome Meiotic DSB Map

The use of *rad50S-*like mutants to map DSBs in budding and fission yeast has resulted in a DSB landscape that is dominated by DSB hot spot clusters separated by 50–200-kb cold regions [32,34,39,40]. In these maps, DSBs are reduced or absent from large (∼40 kb) regions at chromosome ends and near centromeres [32,34]. Although a similar DSB pattern is seen in *rad50S* mutants in this study (Figures 1 and 2 and Figure S4), a very different DSB map emerges when meiotic ssDNA is analyzed in *dmc1Δ* mutants (Figures 3 and 4 and Figures S1 and S2). About twice as much of the genome displays a significant DSB signal (2× background) in *dmc1Δ* than in *rad50S* (70%–80% versus 35%), and the overall DSB signal in *dmc1Δ* is about twice that seen in *rad50S* (Figure S5 and Table S7). This conclusion is confirmed by Southern blot studies and by pulsed-field gel analysis of DSBs on normal chromosomes *III*, where DSBs are substantially more as frequent in *dmc1Δ* than in *rad50S* (Figure 1). As a consequence, the S. cerevisiae genome can no longer be described as being composed of large DSB “hot” and “cold” regions. Instead, recombination initiation events are more broadly distributed, with the majority (>70%) of loci being within 2.5 kb of DSB hot spots with detectable break frequencies (Figure 4B).

Because of the increased number of DSB hot spots detected in *dmc1Δ* mutants, it will be important to revisit the sequence, chromatin and chromosome context determinants of DSB hot spots described from a subset of *dmc1Δ-rad50S* conserved DSBs hot spots [32,33,35,42,59–61]. Our current analysis is of insufficient resolution to test suggestions involving chromatin structure or specific sequences. The increased number and intensity of DSB in *dmc1Δ* relative to *rad50S* also makes it important to revisit previous studies that used *rad50S-*like mutants to examine the influence of factors such as global transcription regulators and chromatin modifiers on meiotic DSB locations and levels [35,38,62,63]. These studies examined only a subset of the DSBs that are formed during normal meiosis, and effects detected in these studies might have involved factors that impact the nonphysiological under-representation of some DSBs in *rad50S*-like mutants.

Replication timing is one factor that has been shown to affect DSB levels in *rad50S*-like mutants, but not in wild type or *dmc1Δ* ([19], see Figure 1). It is unlikely that differences in replication timing can account for all of the differences that we observe between *dmc1Δ* and *rad50S* DSB distributions. Mitotic replication timing patterns [64,65] do not correspond well with genome-wide patterns of concordance and discordance between *dmc1Δ* and *rad50S* (unpublished data). Furthermore, concordant and discordant DSB sites can be found within a single 10-kb region, a distance that is considerably less than the distances (∼40 kb) over which substantial differences in replication timing are observed (see Figure S5). It will be of considerable interest to identify the factors that determine why, at many sites, DSBs are recovered frequently at in *dmc1Δ* and wild type, but infrequently in *rad50S*-like mutants. While we have assumed that this discordance reflects a failure to form DSBs at some sites in *rad50S*-like mutants, it is possible that breaks are formed transiently at some sites but rapidly repaired without resection, perhaps by reversal of the initial Spo11 cleavage reaction (e.g., [66]).

### DSBs Form near Centromeres and Chromosome Ends

Previous studies using *rad50S-*like mutants suggested that DSBs are largely absent from regions within 50 kb of chromosome ends and in ∼40 kb regions around centromeres [33,34,62], consistent with the need to exclude recombination from centromeres and chromosome ends to prevent chromosome segregation dysfunction [2,67,68]. The current analyses indicate that these DSB-cold regions are considerably smaller than previously suggested. While repression of meiotic recombination has been clearly documented for the chromosome *III* centromere ([69–71], T-C Wu, M. Lichten, unpublished data), our data indicate that this may not be true for all other chromosomes. Similarly, whereas the first ∼20 kb from chromosome ends are DSB cold, the following ∼30-kb display substantial DSB activity in single-copy sequences, a finding consistent with recent studies of crossing-over near chromosome ends (A. Barton, D. Kaback, personal communication; S. Chen, J. Fung, personal communication). It should also be noted that ectopic meiotic exchange is reported to occur frequently between repeated genetic elements immediately adjacent to chromosome ends [72], implying that DSBs can form in these elements as well. These repeated elements were not included in the microarrays used in our analysis.

### *DMC1* and *SPO11* Dependence of Meiotic Recombination

Previous studies have shown that most meiotic recombination is initiated by Spo11-catalyzed DSBs [3], but this has not been confirmed on a genome-wide basis. In addition, studies at individual test loci [17,45,73] showed that meiotic DSB repair can occur in a Dmc1-dependent, Rad51-independent manner, but do not exclude the possibility that repair in other regions is Dmc1-independent and Rad51-dependent. We find no regions where ssDNA enrichment values in *rad51Δ dmc1Δ* are more than 2-fold greater than those in *dmc1Δ* (Table S1, Figure S3). This confirms Dmc1 as a critical strand-exchange activity for meiotic DSB repair in all single-copy regions. This conclusion does not exclude an important role for Rad51 in meiotic recombination, but the observation of significantly lower levels of meiotic ssDNA in a *rad51Δ* single mutant (Tables S1 and S7 and Figure S4) suggests that substantial meiotic DSB repair can occur in its absence during budding yeast meiosis, consistent with reports of meiosis-induced activities that inhibit Rad51 [74]. Similarly, Spo11-independent lesions such as nicks and/or DSBs, if processed to form substrates for strand invasion, should be detected as ssDNA in the absence of Dmc1 and Rad51. We detected no meiosis-specific ssDNA enrichment in a *spo11-Y135F dmc1*Δ strain, either at three DSB hot spots (Figures 2 and 3) or in the genome as a whole (Figure S2 and Table S1). This observation strongly supports the previous conclusions that Spo11-catalyzed DSBs initiate the vast majority of, if not all, meiotic recombination events.

### Implications for Recombination Patterns in Other Organisms

The general absence of large DSB-hot and DSB-cold regions that we observe in S. cerevisiae is consistent with the relatively uniform distribution of estimated crossover activity per unit distance over large intervals in the budding yeast genome as a whole [41]. It stands in contrast to the highly punctuated crossover maps, with pronounced recombination hot spots separated by 0.1–1 Mb of relatively inactive regions, in several multicellular organisms, in particular in those inferred from human linkage disequilibrium data [75–80]. Comparisons between these highly punctuated recombination maps and the DSB map from yeast *rad50S*-like mutants have suggested that the molecular-level yeast picture might be an appropriate paradigm for what occurs at the molecular level in other organisms; however, the absence of marked “hot” and “cold” regions in the new yeast DSB map indicates otherwise. If distance comparisons are made in terms of overall chromosome length, then the distribution of recombination hot spots in higher eukaryotes more closely resembles the relatively uniform distribution of DSB hot spots in S. cerevisiae. In addition, this latter distribution is consistent with the relatively uniform distributions of early cytological structures that are presumed to reflect early interhomolog interactions at the onset of crossover and noncrossover recombination events [81–84]. In particular, the use of DSB-mediated interhomolog interactions at sites of random collision to drive homolog pairing during early meiosis 1 prophase [57,85], would be expected to select for a relatively uniform DSB distribution where the distance between hot spots scaled with chromosome size.

## Materials and Methods

### Yeast strains and cultures.

All S. cerevisiae strains used (Table S6) are isogenic to the SK1 background [86]. All markers were introduced by transformation and by genetic crosses between transformants. Genetic distances were determined by tetrad analysis, using the Stahl lab calculator, available at http://rd.plos.org/pbio.0050324.

### Meiotic DNA preparation and Southern blot analysis.

To minimize ssDNA formation during purification, we used direct lysis in phenol/chloroform and digested DNA with restriction enzymes to produce fragments similar in size to those produced in chromatin immunoprecipitation procedures. Cells were harvested from 25 ml of a sporulation culture 5 h after initiation of sporulation, which was identified as a time when most DSB formation is complete by Southern blot–based studies of *dmc1* mutants, as well as by cumulative curve analysis [87] of Southern blots of wild-type strains (unpublished data, see also Figure 6 and Figure S7). For a detailed protocol, see Protocol S1. Pulsed-field gels used DNA prepared in agarose plugs as described [38]. Agarose gel electrophoresis, pulsed-field gel electrophoresis, Southern blotting, and hybridization with radioactive probe were as described [19]. Radioactive signal on filters was detected and quantified using a Fuji FLA 5100 scanner and ImageGauge 4.1 software. For pulsed-field gels in Figure 1, densitometry traces were divided into ∼720 bins, and DSB frequencies were corrected to account for signal reduction due to coincident cutting in the bin in question and in probe-proximal bins, using the following formula:

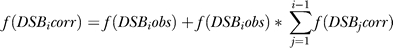
where *DSB_i_ =* DSB signal in bin *i*. This correction assumes that DSBs are distributed randomly with respect to each other along chromosomes, so that for a chromosome with a DSB at locus *i*, the likelihood of additional DSBs between *i* and the chromosome end is the same as the likelihood of DSBs between *i* and the chromosome end in an unselected population of chromosomes.

### Spo11 ChIP and meiotic ssDNA enrichment on BND cellulose.

ChIP of hemagglutinin (HA)-tagged Spo11 from a *rad50S* strain was performed as described with only minor modifications, using cells from a culture 5 h after initiation of sporulation [33,34]. ssDNA enrichment on BND cellulose (Sigma) used batch purification [88]; for detailed protocols, see Protocol S1. Enrichment of DSB-associated DNA was estimated by quantitative PCR (details in Protocol S1).

### Microarray hybridization and background normalization.

Input and ssDNA-enriched material from BND cellulose fractionation and Spo11-ChIP fractions and whole-cell extracts from Spo11 immunoprecipitates were amplified using a previously described random priming amplification procedure with minor modifications [33] (details in Protocol S1).

Background normalization of fluorescence signals in each channel was performed using a subset of probes for which the presence of meiotic ssDNA is unlikely (Table S2). Southern blot analyses and fine-structure mapping of DSB hot spots have shown that meiotic DSBs are generally absent from protein coding regions [32,55]. Given an average shear size of 1 kb in Spo11 ChIP and 1 kb resection tract in DNA from *dmc1Δ* mutants, we reasoned that the hybridization signal of array elements located at least 2 kb from the 3′ and 5′ ends of protein coding sequences are likely to represent background. The median fluorescence intensity of elements meeting this criteria, selected from the largest open reading frames in the budding yeast genome (294 array elements representing 0.7% of the total number of elements) was used to normalize the fluorescence intensity for each spots in each channel. For each hybridization, we then calculated the ratio of the background-normalized Cy-5 (experimental) channel fluorescence versus the average of five independent background-normalized hybridizations with Cy-3 labeled genomic DNA. All experiments were performed in duplicate from independent cultures; the data presented in Table S1 are the average of the two independent ratios for each mutant background.

### Microarray data analysis.

MATLAB (v. 7.4.0), Microsoft Excel (v. 11.3.3), and Graphpad Prism (v. 4.0b) were used for computational and statistical analysis; program code will be supplied upon request. DSB hot spots were identified in microarray hybridization data using a MATLAB implementation of the PeakFinder program initially developed for PCR product arrays [89]. A seven-element running average was first applied to background-normalized ratios, and DSB peaks at different threshold levels were identified using the first derivative of this de-noised data. A seven-element running average results in a loss of some individual peaks, especially in gene-dense regions, but this window size is the smallest that avoids peak doubling, as the random labeling protocol used here results in two closely-spaced peaks flanking each DSB site. Peak coordinates determined for various thresholds were then used to calculate interpeak distances and the fraction of genome within a given distance from a DSB site.

## Supporting Information

Figure S1 Comparison of DSB Signals in *dmc1*Δ and *rad50S* Mutants Distribution of background-normalized, unsmoothed Spo11 ChIP ratios from *rad50S* (orange) and BND cellulose–enriched ssDNA ratios from *dmc1*Δ (blue) and *spo11Y135F dmc1Δ* (red). Black dot indicates centromeres. For each mutant, data (Table S1) are the average of two independent experiments.
(522 KB PDF)

Figure S2 Comparison of DSB Signals in *dmc1*Δ and *rad51*Δ *dmc1*Δ Mutants Distribution of BND cellulose–enriched ssDNA ratios from *dmc1*Δ (blue) and *rad51*Δ *dmc1*Δ (pink). Data (Table S1) are the average of two independent experiments.
(531 KB PDF)

Figure S3 Comparison of DSB Signals in *dmc1*Δ and *spo11-Y135F dmc1*Δ Mutants Distribution of BND cellulose–enriched ssDNA ratios from *dmc1*Δ (blue) and *spo11-Y135F dmc1*Δ (red). For each mutant, data (Table S1) are the average of two independent experiments.
(502 KB PDF)

Figure S4 Comparison of DSB Signals in *dmc1Δ* and *rad51Δ* Mutants Distribution of BND cellulose–enriched ssDNA ratios from *dmc1*Δ (blue) and *rad51*Δ (purple). Data (Table S1) are the average of two independent experiments for *dmc1Δ* and a single experiment for *rad51Δ*.
(530 KB PDF)

Figure S5 Discordance between *dmc1Δ* and *rad50S* DSB Signals Distribution of background-normalized and smoothed (see Materials and Methods) Spo11 ChIP ratios from *rad50S* (orange) and BND cellulose–enriched ssDNA ratios from *dmc1*Δ (blue). Colored squares denote array elements where the *dmc1Δ* signal in two successive elements is at least two times (orange), three and a half times (red), and five times (purple) the corresponding *rad50S* signal, respectively. The number of array elements and fraction of genome with *dmc1Δ*/*rad50S* signal ratios are as follows: >2, 16,290, 40%; >3.5, 3,098, 7.6%; >5, 577, 1.4%. DSB-cold regions are defined as regions of ≥33 array elements (∼10 kb) where all *rad50S* signals are less than twice background (gray bars, 260 regions, 4.8 Mb, 40% of genome) or where both *dmc1Δ* and *rad50S* signals are less than twice background (black bars, 370 kb, 3% of genome). No regions exist where *dmc1Δ* but not *rad50S* signals are less that twice background.
(854 KB PDF)

Figure S6 Southern Blot Analysis of DSBs in *rad50S*, *dmc1*Δ and *spo11-Y135F*
DSBs were examined in the following regions: (A) *YDR186c*-*YDR188w* (chr *IV*); (B) left-hand end of chr *XIII*; (C) near centromere of chr *XI*. DNA purified from *spo11-Y135F* (MJL3096), *dmc1*Δ (MJL3095), and *rad50S* (MJL1083) was displayed on Southern blots after digesting with the following enzymes: (A) *Pst*I; (B) *Pvu*I, and (C) *Sac*I. Probes are PCR products from the indicated open reading frames (*). DSB frequencies (% total lane signal) are indicated to the right of each blot for *rad50S* (orange) and *dmc1*Δ (blue). DNA length standards contain a *Bst*EII digest of phage λ DNA (A) and a *Hin*dIII digest of phage λ DNA (B and C).
(358 KB PDF)

Figure S7 Southern Blot Analysis of DSBs in Wild Type Analysis of additional discordant regions between *rad50S* and *dmc1Δ* maps. Experimental details are as in Figure 6. (A) DSBs in the *YDL220c* region. Upper left: Southern blot showing DSBs in DNA from wild type, *dmc1Δ*, and *rad50S*. Digest: *Mlu*I. Arrows: open reading frames in the region, top to bottom: *YDL223c*; *YDL222c*; *YDL220c* (probe). Upper right: density trace, normalized to total lane density, of the indicated lanes. Peak densities are in terms of % of total lane density. Lower left: background-normalized average DSB signals from microarrays for the same region. Lower right: DSB timing in wild type, for the DSB peaks indicated in the density trace.
(B) DSBs in the discordant region *YOR347c*. Panels are as indicated in (A). Digest: *Pst*I, Arrows: open reading frames in the region, top to bottom: *YOR349w*; *YOR348c*; *YOR347c* (probe). Lanes containing *dmc1Δ* and *rad50S* samples are from a different gel, and the length of that portion of the image has been adjusted.
(503 KB PDF)

Figure S8 Correlation between DSB Intensity and ssDNA Enrichment Ratios in DNA from *dmc1Δ*
Integrated DSB peak volumes from microarrays are plotted versus corresponding band densities from Southern blots (Figure 5 and Figures S6 and S7). In cases where DSB peaks could not be resolved on array plots, array and blot signals for multibreak region were summed. Regression line formula: *f*(DSB southern blot, %) = 0.026*(DSB microarray signal) + 0.6; *R* ^2^ = 0.79.
(33 KB PDF)

Protocol S1 Supplementary Methods and References (44 KB DOC)

Table S1 Average and De-Noised Ratios of Background-Normalized Fluorescence Average ratios are calculated form background-normalized ratios of two independent experiment for each mutant (Spearman rank correlation analyses of F635 fluorescence signal background normalized are *dmc1*Δ-0.85, *rad51Δ dmc1Δ-*0.86, and *rad50S*-0.59). Smoothed ratios were calculated using a running average of seven consecutive probes.
Agilent index—manufacturer's array element identifier.
Name—array element identity supplied by [53].
Blanks represent elements where data was missing or values could not be calculated.
(3.5 MB TXT)

Table S2 Array Elements Used for Background Normalization Probes associated with the 19 largest genes in the genome (>7 kb) were first selected. To avoid any signal arising from promoter or intergenic regions, probes below 2 kb from the 5' or 3' end of the genes were removed. Column headings are as in Table S1.
(57 KB XLS)

Table S3 ssDNA Enrichment from *dmc1Δ* at the 50 Strongest *rad50S* Hot Spots The 50 highest DSB peaks in the Spo11 ChIP dataset from *rad50S* (Table S5) are compared to corresponding DSB peaks from ssDNA-enriched material from *dmc1Δ*. Because of peak shifting in smoothed data, sometimes adjacent elements are identified as peaks in *rad50S* and *dmc1Δ* data, resulting in different names being assigned.
(22 KB XLS)

Table S4 Comparison of Spo11 ChIP in This Study with That of Borde et al. Average de-noised Spo11 ChIP ratios from this study (Table S1) are given for DSB peaks corresponding to the 50 elements with the strongest DSB signal reported by Borde et al. [34]. Peak locations were identified by running-average smoothing combined with a peak detection algorithm [89] as described in Protocol S1. Columns are, from left to right: element name used by Borde et al.; Spo11 ChIP element signal in Borde et al.; Spo11 ChIP signal at peak array element in this study corresponding to the PCR array element in Borde et al.; and name of peak array element given by Agilent. Asterisks in column 1 denote PCR array elements that flank a single DSB (for example, *YDR187c* and *YDR188w*).
(39 KB XLS)

Table S5 DSB Hot Spots at Two Thresholds DSB peaks were determined at thresholds of 2× and 5× background (see Protocol S1). Peak elements are numbered from left to right on each chromosome. Interpeak distances were calculated using array element midpoints. Normalized peak intensities are from Table S1.
(655 KB XLS)

Table S6 Strains Used in This Study (50 KB DOC)

Table S7 Statistical Analysis of Background-Normalized Array Datasets (75 KB DOC)

### Accession Numbers

The Entrez Protein (http://www.ncbi.nlm.nih.gov/sites/entrez?db=protein) accession numbers for the proteins corresponding to the genes discussed in this paper are: *CDC28* (NP_009718), *CDC6* (NP_012341), *CLB5* (NP_015445), *DMC1* (NP_011106), *MRE11* (NP_013951), *RAD50* (NP_014149), *RAD51* (NP_011021), *recA* (AAQ91336), *SAE2* (NP_011340), *SPO11* (NP_011841), *YCL011c* (NP_009916), *YCR007c* (NP_009933), *YCR011c* (NP_009937), *YCR019w* (NP_009946), *YCR020c* (NP_009947), *YCR022c* (P25620), *YCR045c* (NP_009974), *YCR046c* (NP_009975), *YCR047c* (NP_009976), *YCR048w* (NP_009978), *YCR051w* (NP_009980), *YCR052w* (NP_009981), *YDL220c* (NP_010061), *YGR176w* (P32475), *YIR020c* (P40575), *YLR436c* (NP_013540), *YLR437c* (NP_013541), *YLR438w* (NP_013542), *YLR439w* (NP_013544), *YLR440c* (NP_013545), *YOR347c* (NP_014992). The microarray data used in this paper are deposited at http://www.ncbi.nlm.nih.gov/geo/ with accession number GSE8981.

## Abbreviations

*arsΔ*: *ars305Δ* *ars306Δ* *ars307*
BND: benzoyl naphthoyl DEAE
ChIP: chromatin immunoprecipitation
chr *III-L*: chromosome *III* left arm
DSB: DNA double-strand break
nt: nucleotide
oligo: oligonucleotide
ssDNA: single-strand DNA
