# Correction: Mapping Meiotic Single-Strand DNA Reveals a New Landscape of DNA Double-Strand Breaks in Saccharomyces cerevisiae

**DOI:** 10.1371/journal.pbio.0060104

**Published:** 2008-04-29

**Authors:** Cyril Buhler, Valérie Borde, Michael Lichten

## Abstract

.

Correction for:

Buhler C, Borde V, Lichten M (2007) Mapping meiotic single-strand dna reveals a new landscape of DNA double-strand breaks in Saccharomyces cerevisiae. PLoS Biol 5(12): e324. doi:10.1371/journal.pbio.0050324

Two figure legends refer to the wrong strain of yeast.

In Figure 2, the second sentence of (B) should say: Spo11 enrichment ratios for *rad50S* (MJL2404) were determined from input samples and immunoprecipitates (ChIP).

In Figure 3, the first sentence should say: In each plot, normalized, unsmoothed array signals (average of two experiments) are as follows: orange, Spo11 ChIP from *rad50S* (MJL2404); blue, BND cellulose-enriched ssDNA from *dmc1*Δ (MJL3095); red, BND cellulose-enriched ssDNA from s*po11Y135F dmc1*Δ (MJL 3096).

