# Supplementary material for: Mapping Meiotic Single-Strand DNA Reveals a New Landscape of DNA Double-Strand Breaks in Saccharomyces cerevisiae
Source: PLoS Biol. 2007 Dec 11;5(12):e324. doi: 10.1371/journal.pbio.0050324 (PMC2121111; doi:10.1371/journal.pbio.0050324)
Supplement: Supplementary file 3 [file pbio.0050324.sg003.pdf]

Figure s3

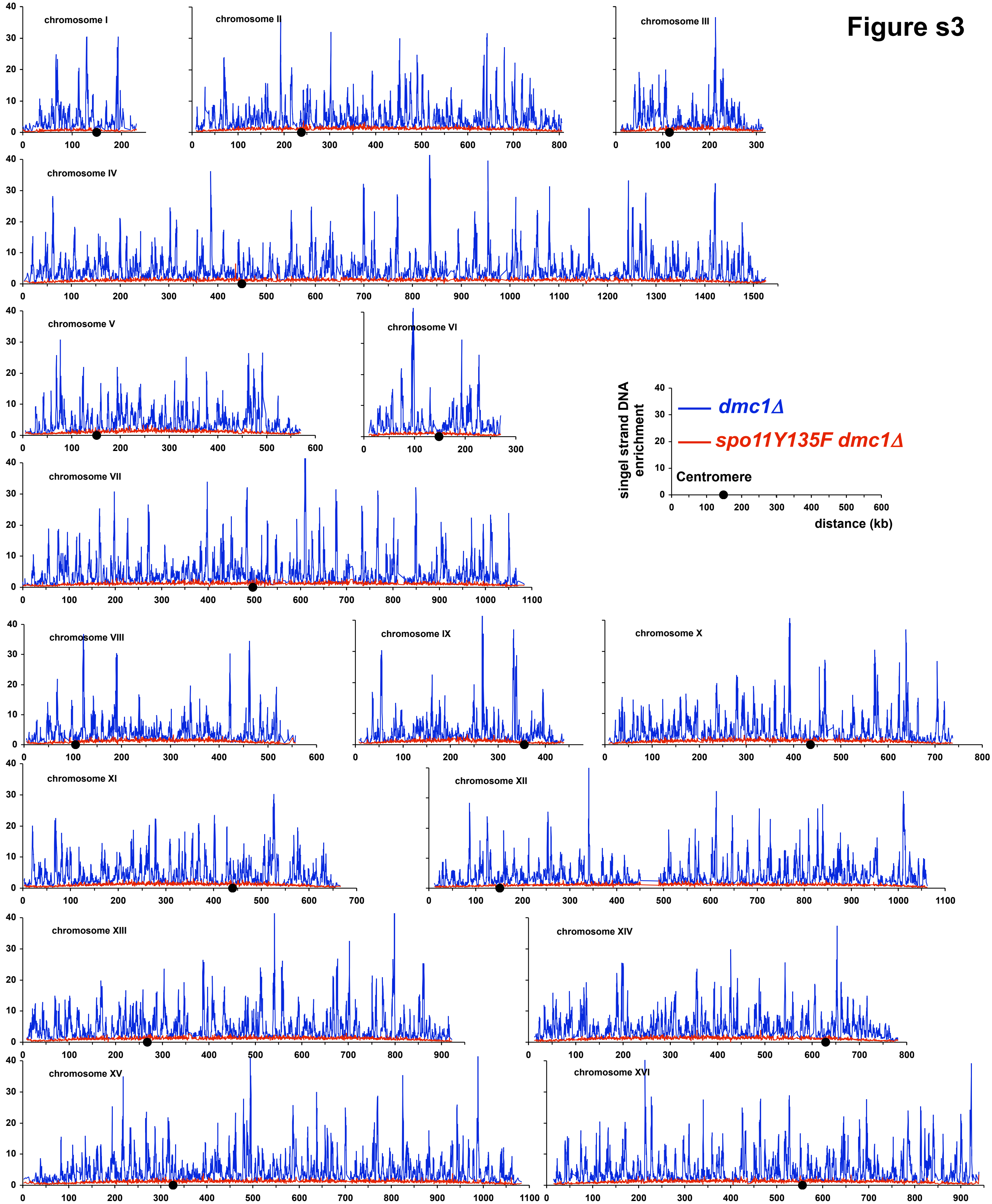
chromosome I
chromosome II
chromosome III
chromosome IV
chromosome V
chromosome VI
chromosome VII
chromosome VIII
chromosome IX
chromosome X
chromosome XI
chromosome XII
chromosome XIII
chromosome XIV
chromosome XV
chromosome XVI
singel strand DNA enrichment
dmc1Δ
spo11Y135F dmc1Δ
Centromere
distance (kb)
