# Supplementary material for: Mapping Meiotic Single-Strand DNA Reveals a New Landscape of DNA Double-Strand Breaks in Saccharomyces cerevisiae
Source: PLoS Biol. 2007 Dec 11;5(12):e324. doi: 10.1371/journal.pbio.0050324 (PMC2121111; doi:10.1371/journal.pbio.0050324)
Supplement: Supplementary file 6 [file pbio.0050324.sg006.pdf]

Figure s6

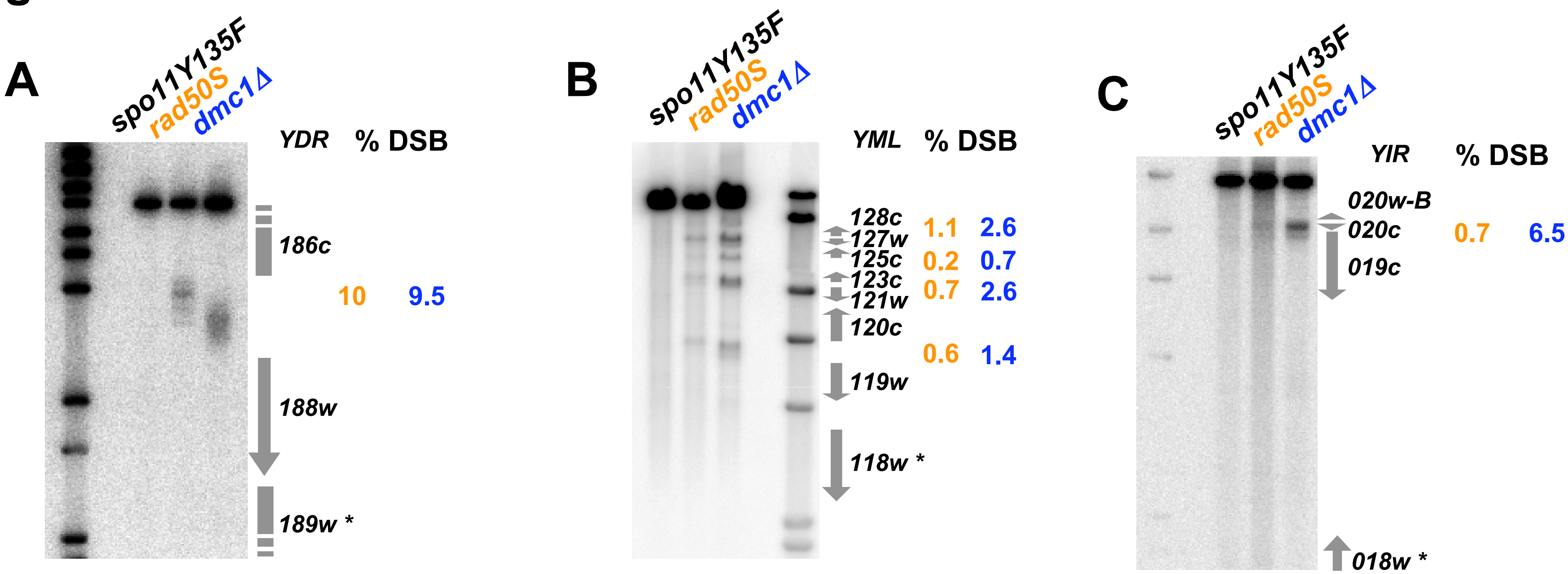

A
spo11Y135F
rad50S
dmc1Δ
YDR
% DSB
186c
10
9.5
188w
189w *
B
spo11Y135F
rad50S
dmc1Δ
YML
% DSB
128c
127w
125c
123c
121w
120c
119w
118w *
1.1
2.6
0.2
0.7
0.7
2.6
0.6
1.4
C
spo11Y135F
rad50S
dmc1Δ
YIR
% DSB
020w-B
020c
019c
018w *
0.7
6.5
