# Supplementary material for: Mapping Meiotic Single-Strand DNA Reveals a New Landscape of DNA Double-Strand Breaks in Saccharomyces cerevisiae
Source: PLoS Biol. 2007 Dec 11;5(12):e324. doi: 10.1371/journal.pbio.0050324 (PMC2121111; doi:10.1371/journal.pbio.0050324)
Supplement: Supplementary file 7 [file pbio.0050324.sg007.pdf]

Figure s7

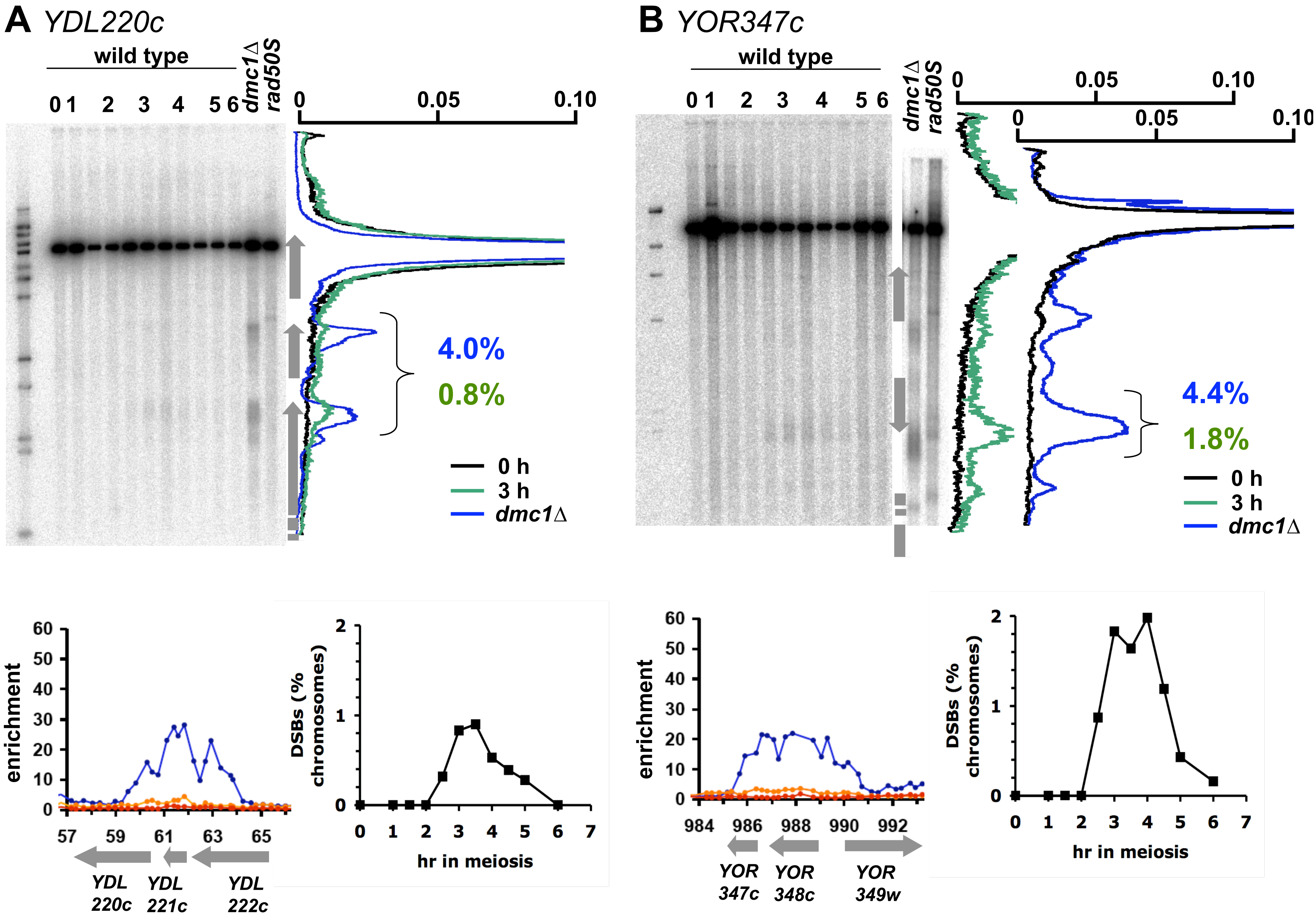

A YDL220c
wild type
0 1 2 3 4 5 6
dmc1Δ
rad50S
0
0.05
0.10
4.0%
0.8%
0 h
3 h
dmc1Δ
B YOR347c
wild type
0 1 2 3 4 5 6
dmc1Δ
rad50S
4.4%
1.8%
enrichment
60
50
40
30
20
10
0
57
59
61
63
65
YDL 220c
YDL 221c
YDL 222c
DSBs (% chromosomes)
2
1
0
1
2
3
4
5
6
7
hr in meiosis
984
986
988
990
992
YOR 347c
YOR 348c
YOR 349w
