# Supplementary material for: Mapping Meiotic Single-Strand DNA Reveals a New Landscape of DNA Double-Strand Breaks in Saccharomyces cerevisiae
Source: PLoS Biol. 2007 Dec 11;5(12):e324. doi: 10.1371/journal.pbio.0050324 (PMC2121111; doi:10.1371/journal.pbio.0050324)
Supplement: Supplementary file 8 [file pbio.0050324.sg008.pdf]

Figure S8

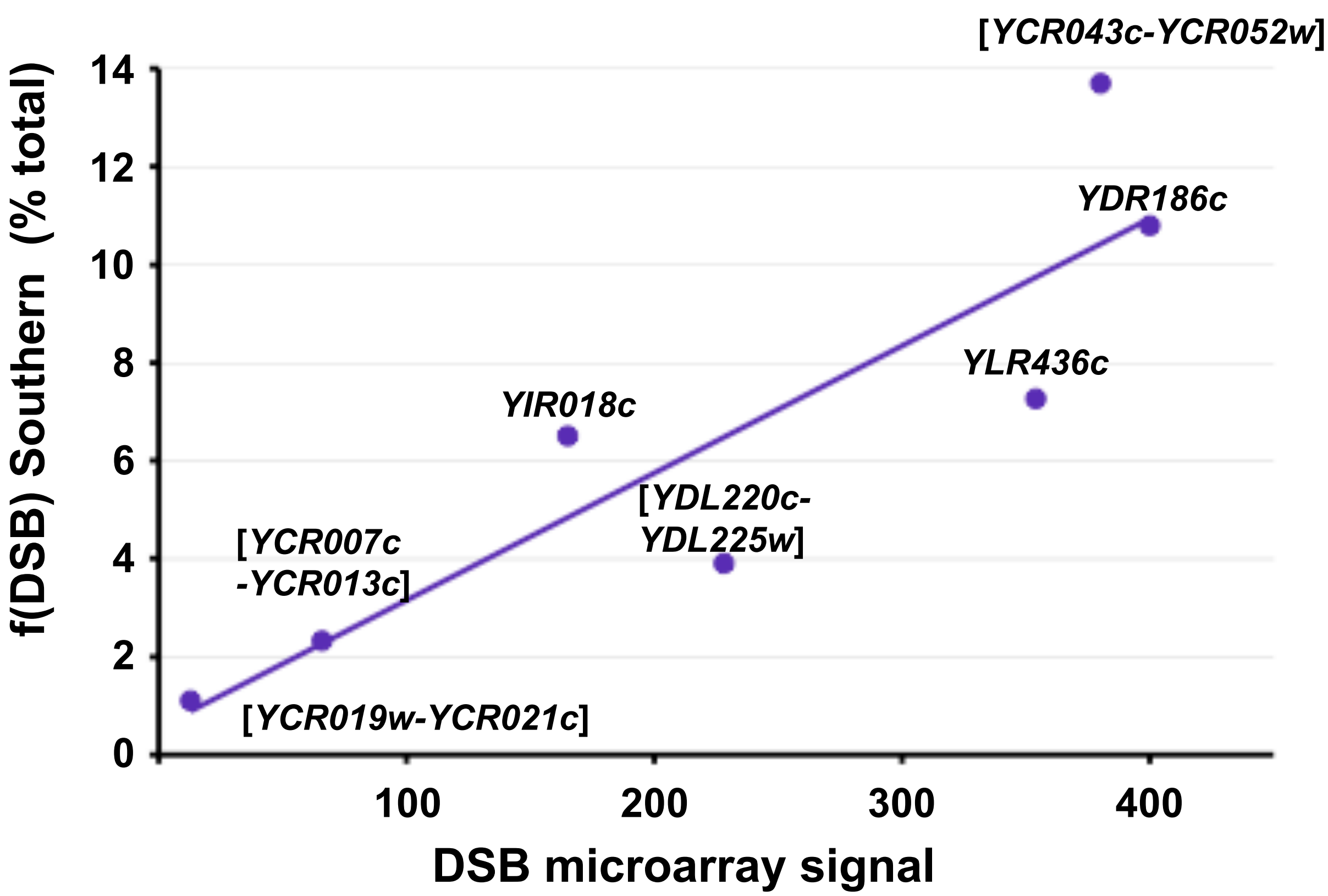

f(DSB) Southern (% total)
0
2
4
6
8
10
12
14
100
200
300
400
DSB microarray signal
[YCR043c-YCR052w]
YDR186c
YLR436c
YIR018c
[YDL220c-
YDL225w]
[YCR007c
-YCR013c]
[YCR019w-YCR021c]
