# Supplementary material for: Mapping Meiotic Single-Strand DNA Reveals a New Landscape of DNA Double-Strand Breaks in Saccharomyces cerevisiae
Source: PLoS Biol. 2007 Dec 11;5(12):e324. doi: 10.1371/journal.pbio.0050324 (PMC2121111; doi:10.1371/journal.pbio.0050324)
Supplement: Supplementary file 9 [file pbio.0050324.sd001.doc]

**Protocol S1. Supplementary Methods**

**Meiotic DNA preparation and Southern blot analysis.** Sporulation was performed at 30˚C as described [1]. Cells were harvested from 25 ml of a sporulation culture 5 hr after initiation of sporulation, a time cumulative curve analysis [2] identified as a time when most DSB formation is complete (data not shown). Cells were resuspended in 0.5 ml of 1.2 M sorbitol, 50 mM KPO_4_ pH 7.5, EDTA 10 mM, 1% 2-mercaptoethanol, 0.25 mg/ml of zymolyase 100T (MP Biomedical) and incubated at room temperature for 15-20 min until cells were ~ 90% spheroplasted. Spheroplasts were harvested and resuspended in 0.5 ml of 100 mM NaCl, 50 mM Tris-HCl pH 8.0, 50 mM EDTA. For strains other than *rad50S*, 1 volume of phenol/chloroform/isoamyl alcohol (25:24:1) pH 7.5 was added and DNA was slowly extracted on a rotary wheel for 15 min. Aqueous and organic phases were separated by centrifugation at 12000g for 5 minutes, and the aqueous phase was extracted with chloroform/isoamyl alcohol (24:1) and centrifuged as before. Nucleic acids were precipitated by addition of 1/10 vol of 5M NaCl and 1 volume of isopropanol. The nucleic acid pellet was resuspended in 300 µl of TE (10 mM Tris-HCl, 1 mM EDTA, pH 8.0), 0.1 mg/ml DNase-free RNase A (Roche) and incubated for 30 minutes at 37 ˚C. DNA was precipitated by addition of 30 µl of 3M sodium acetate (pH 5.2) and 200 µl isopropanol, and the pellet was washed with 70% (v/v) ethanol and resuspended in TE. DNA was prepared from *rad50S* strains as described [3].

**Meiotic single strand DNA enrichment on BND cellulose.** 10 µg of genomic DNA prepared by direct phenol/chloroform extraction (see above) was digested with *Hind*II and *Ssp*I (Roche) for 4 hours (2 units per µg of DNA), which results in a predicted median fragment size of 400 bp. 100 µl of BND cellulose slurry (corresponding to 50 µl of packed matrix) was equilibrated in TEN buffer (Tris 10 mM, EDTA 1 mM, 1M NaCl pH 8.0) by three successive centrifugations (1 min, 1000g) and resuspension in 450 µl TEN buffer to remove particles. NaCl (5M) was added to restriction digests to 1M final, and digested material was combined with the packed BND cellulose. After 10 min incubation at room temperature with occasional mixing, matrix plus bound DNA was isolated by five successive centrifugations (1000g, 1 min) with intervening resuspension in 450 µl TEN. Single strand-enriched DNA was eluted in 5 similar washes of 450 µl of TEN + 1.7% (w/v) caffeine (Sigma). Caffeine-eluted fractions were combined, remaining BND cellulose was removed using a glass filter spin columns (Harvard Apparatus), and pooled fractions concentrated and desalted by 5 successive buffer exchanges with 500 µl TE, using a Microcon YM-30 spin-filter. Caffeine removal was monitored by measuring flow-through absorbance at 270nm.

**Quantitative PCR.** The following primers were used to measure relative number of genome equivalents of target sequences in DNA samples:

rDNA--	rDNAup 5'-CTGATGTCTTCGGATGGATTTGAG-3'

rDNAdw 5'-TTTCCTCTGGCTTCACCCTATTC-3'

*YCR047c*--	qBUD23up 5'-TATGTCGTCCACCTGGTCGTCG-3'

qBUD23dw 5'-TCCTAAACAGCGGTTGATGAGG-3'

*YGR176w*--	qERG1up 5'-CAGTCATACCACCACCAGTCAATG-3'

qERG1dw 5'-GCCAAACTCCTACTTGCCAGC-3'

Primers specific to rDNA were used to measure background DNA not associated with meiotic DSBs. Primers close to the *YCR047c* and *YGR176w* promoter regions were used to measure recovery of DSB-associated sequences. Approximate distances from PCR products to the nearest DSB are 1.2 kb for *YGR176w* and 0.6 kb for *YCR047c*. A dilution series of genomic DNA (S656) was used to establish a standard curve, and enrichment was calculated as the ratio of target locus genome equivalents/rDNA genome equivalents in samples. qPCR was performed in an IQcycler (BioRad), using 0.2 µM primers, iQ SYBR Green Supermix (BioRad) and the following PCR program: 95 ˚C for 10 sec; 57 ˚C for 10 sec; 72 ˚C for 10 sec; 40 cycles. Only PCR reactions with amplification efficiencies between 90% and 100% were used.

**Microarray hybridization.** A modification of the random priming amplification procedure was used (original protocol at http://cat.ucsf.edu/resources/index.html). Two successive primer extensions (Round A) were performed on DNA samples using T7 polymerase (Sequenase, USB) and primer A (5'-GTTTCCCAGTCACGATCNNNNNNNNN-3'), the first 17 nt of which are absent from *S. cerevisiae* genome. For ssDNA-enriched material, the first extension was performed without prior denaturation to select for the single strand templates; for ChIP samples both extensions were preceded by denaturation. The following program was used: 10 °C to 37 °C, ramp time-8 min; 37 °C for 8 min; 94 °C for 2 min; 10°C for 5 min (sequenase added); 10 °C to 37 °C, ramp time-8 min; 37°C for 8 min; 24 ˚C for 10 sec. All amplifications included a second round (Round B) using *Taq* polymerase (Promega) and primer B (5'-GTTTCCCAGTCACGATC-3') using the following program: 95 ˚C for 30 sec; 47 ˚C for 30 sec; 72 ˚C for 2 min; for 15, 18, 21, 24 or 27 cycles. The extent of amplification was assayed by displaying 5% of each reaction on a 1.5% agarose gel and staining with SYBR green (Molecular Probes). Samples with the least number of amplification cycles showing detectable product were selected, and primer dimmers were removed by two successive filtration/wash cycles using Microcon YM-100 spin filters (Millipore) and 500 µl of TE. Aminoallyl-dUTP was then incorporated by PCR (Round C) using the same program and primers as in Round B, but a 3/2 ratio of aminoalyl dUTP/dTTP and 15 units of *Taq* polymerase per reaction. Amplified material was purified by two successive filtration/wash cycles using Microcon YM-100 spin filters (Millipore) 500 µl of 10 mM Na_2_CO_3_ pH 8.9. qPCR was performed on final amplification products to confirm the maintenance of enrichment levels present in the starting material. 2µg of amplified material from Round C was labeled with Cy-5 monoreactive dye (GE Healthcare) and 500 ng of similarly amplified control material (BND cellulose input or whole cell extract) was labeled with Cy-3 (GE Healthcare) in 50 mM Na_2_CO_3_ pH 8.9 for 1 hour in the dark. Unincorporated dye was removed by 3 successive washes with 400 µl MES 50 mM pH 7.2 (Sigma) using Microcon YM-50 spin filters (Millipore). Dye incorporation was estimated using by measuring dye adsorbance at 550 nm (Cy-3) and 650 nm (Cy-5) in final products. Typical substitution levels were 1 labeled nt/20 nt. A detailed protocol will be supplied upon request. 500 ng of each labeled sample were hybridized to an Agilent 44k yeast whole genome oligonucleotide array (Agilent, G4486A) for 17 hours at 60˚C in the 1X hybridization buffer supplied by Agilent. Slides were washed for 5 min in 6X SSPE (SSPE = 150 mM NaCl, 10 mM M Na_2_HPO_4_, 1 mM EDTA, pH 7.4), 0.05% (w/v) N-lauroylsarcosine, once in 0.06X SSPE for 5 minutes, and were then rinsed in the stabilizing and drying solutions supplied by Agilent. Slides were immediately scanned using an Axon 4000B scanner set at 5 µm resolution with automatically adjusting laser PMT values to achieve a maximum fluorescence saturation of 0.005%. Fluorescence data was extracted using GenePix 6.0 (Axon) software. For each channel, features were filtered according to the following criteria. First, all spots with a diameter less than 50 µm were removed. The background fluorescence in each channel was then calculated using the mean fluorescence value of 315 empty array elements. All array elements with fluorescence less than background + 2 standard deviations were removed, as were all array elements with a signal to background ratio (signal +background)/ background) less than 3. These filtering conditions identified a set of array elements that were consistently removed from independent hybridizations with genomic DNA, sheared by sonication to an average size of 1 kb and labeled as above with Cy-3. These elements most likely represent sequences present in the reference strain but absent from SK1. 515 of a total of 41282 array elements met this criterion and were removed from subsequent analyses.

**References**.

1. Goyon C, Lichten M (1993) Timing of molecular events in meiosis in *Saccharomyces cerevisiae*: stable heteroduplex DNA is formed late in meiotic prophase. Mol Cell Biol 13: 373-382.

2. Padmore R, Cao L, Kleckner N (1991) Temporal comparison of recombination and synaptonemal complex formation during meiosis in *S. cerevisiae*. Cell 66: 1239-1256.

3. Borde V, Wu TC, Lichten M (1999) Use of a recombination reporter insert to define meiotic recombination domains on chromosome *III* of *Saccharomyces cerevisiae*. Mol Cell Biol 19: 4832-4842.
