# Supplementary material for: Mapping Meiotic Single-Strand DNA Reveals a New Landscape of DNA Double-Strand Breaks in Saccharomyces cerevisiae
Source: PLoS Biol. 2007 Dec 11;5(12):e324. doi: 10.1371/journal.pbio.0050324 (PMC2121111; doi:10.1371/journal.pbio.0050324)
Supplement: Supplementary file 10 [file pbio.0050324.st006.doc]

**Table S6 Strains used in this study.**

Name	Genotype*

S656	*MATa leu2-R arg4-bgl nuc1∆::LEU2*

MJL1083	*MATα rad50-K81I::URA3*

*MATa rad50-K81I::URA3*

MJL1578	*MATa leu2 arg4-bgl nuc1∆::LEU2*

*MATα leu2 arg4-bgl nuc1∆::LEU2*

MJL2306	*MATα leu2-K arg4-nsp,bgl ∆ade2 sae2::KanMX6*

*MATa leu2-R arg4-nsp,bgl ∆ade2 sae2::KanMX6*

MJL2404	*MATα leu2 SPO11-3HA-His6::KanMX4 rad50-K81I::ura3::LEU2 nuc1∆::LEU2*

*MATa leu2 SPO11-3HA-His6::KanMX4 rad50-K81I::ura3::LEU2 nuc1∆::LEU2*

MJL2529	*MATα leu2-K ars305∆ ars306∆ ars307-T108569G arg4-nsp,bgl sae2::KanMX6*

*MATa leu2-R ars305∆ ars306∆ ars307-T108569G arg4-nsp,bgl sae2::KanMX6*

MJL2560	*MATα leu2-K arg4-nsp,bgl ∆ade2 dmc1::LEU2*

*MATa leu2-R arg4-nsp,bgl ∆ade2 dmc1::LEU2*

MJL2683	*MATa leu2-R ars305∆ ars306∆ ars307-T108569G* *arg4-nsp,bgl dmc1::LEU2*

*MATα leu2-K ars305∆ ars306∆ ars307-T108569G arg4-nsp,bgl dmc1:: LEU2*

MJL3095	*MATα leu2 arg4 nuc1∆::LEU2 dmc1∆::LEU2*

*MATa leu2 arg4 nuc1∆::LEU2 dmc1∆::LEU2*

MJL3096	*MATα leu2 arg4 nuc1∆::LEU2 dmc1∆::LEU2 spo11-Y135F::URA3*

*MATa leu2 arg4 nuc1∆::LEU2 dmc1∆::LEU2* *spo11-Y135F::URA3*

MJL3236	*MATα leu2-K cha1::KanMX ars305∆ ars306∆ ars307-T108569G arg4-nsp,bgl*

*MATa LEU2	CHA1	ars305∆ ars306∆ ars307-T108569G	ARG4*

MJL3237	*MATα leu2-K cha1::KanMX arg4-nsp,bgl*

*MATa LEU2	CHA1	ARG4*

MJL3271	*MATα leu2	ARG4	nuc1∆::LEU2 rad51::KanMX4*

*MATa leu2 arg4-bgl nuc1∆::LEU2 rad51::KanMX4*

MJL3272	*MATα leu2 arg4 nuc1∆::LEU2 dmc1∆::LEU2 rad51::KanMX4*

*MATa leu2 arg4 nuc1∆::LEU2 dmc1∆::LEU2 rad51::KanMX4*

MJL3274	*MATα leu2 arg4-bgl nuc1∆::LEU2 spo11-Y135F::URA3 dmc1∆::LEU2 rad51::KanMX4*

*MATa leu2 arg4-bgl nuc1∆::LEU2 spo11-Y135F::URA3 dmc1∆::LEU2 rad51::KanMX4*

MJL3275	*MATα leu2 arg4 nuc1∆::LEU2 spo11-Y135F::URA3 dmc1∆::LEU2*

*MATa leu2 arg4 nuc1∆::LEU2 spo11-Y135F::URA3 dmc1∆::LEU2*

*All strains are isogenic to the SK1 background and are homozygous for the following markers: *ura3 lys2 ho::LYS2*.
