# Supplementary material for: Mapping Meiotic Single-Strand DNA Reveals a New Landscape of DNA Double-Strand Breaks in Saccharomyces cerevisiae
Source: PLoS Biol. 2007 Dec 11;5(12):e324. doi: 10.1371/journal.pbio.0050324 (PMC2121111; doi:10.1371/journal.pbio.0050324)
Supplement: Supplementary file 11 [file pbio.0050324.st007.doc]

**Table S7. Statistical Analysis of Background Normalized Array Datasets.**

|  | *dmc1* | *dmc1 rad51* | *rad51* | *rad50S* | *spo11-Y135F* |
| --- | --- | --- | --- | --- | --- |
| Number of non zero elements* | 40762 | 40759 | 40684 | 40766 | 40739 |
| Mean element signal ** | 4.6 ± 4.4 | 5.1 ± 4.3 | 2.1 ± 1.3 | 2.2 ± 2.3 | 1.1 ± 0.4 |
| Skew † | 2.80 | 3.29 | 2.30 | 6.81 | 0.51 |
| Median element signal | 3.1 | 3.8 | 1.8 | 1.6 | 1.1 |
| Maximum element signal | 60 | 65 | 18 | 66 | 6.5 |
| Mean top 100 elements | 36 | 36 | 11 | 28 | 3 |
| Fraction with signal >2 | 0.71 | 0.80 | 0.43 | 0.35 | 0.03 |
|  |  |  |  |  |  |
| Mean background signal†† | 1.2 ± 0.6 | 1.2 ± 0.6 | 1.1 ± 0.5 | 1.1 ± 0.4 | 1.1 ± 0.4 |
| Total array signal-background | 1.3 x 10^5^ | 1.6 x 10^5^ | 4.1 x 10^4^ | 4.1 x 10^4^ |  |
| DSBs/haploid genome††† | 34 | 42 |  | 11 |  |
|  |  |  |  |  |  |
| Among elements with *dmc1∆* signal > 1.5 | |  |  |  |  |
| Fraction with signal > *dmc1∆* |  | 0.70 | 0.06 | 0.07 | 0.01 |
|  |  |  |  |  |  |
| Among elements with *dmc1∆ rad51∆* signal > 1.5 | | |  |  |  |
| Fraction with signal > *dmc1∆ rad51∆* | |  | 0.05 | 0.05 | 0.00 |

*Number of elements with hybridization signal above slide background. Only these elements were considered in statistical analysis

** Mean ± standard deviation

† Deviation of distribution from symmetry around the mean,

$$\frac{n}{(n-1)(n-2)}\sum\left( \frac{\left( xj-\overline{x} \right)}{s} \right)^{3}$$

where n = number of elements, x_j_ = individual elements, ‾‾x = mean element signal, s = standard deviation.

†† Average signal of the 294 array elements chosen for background normalization (see Experimental Procedures and Table S2).

††† Calculated from the total DSB signal, using the regression formula f(DSB southern blot, %) = 0.026*(DSB microarray signal) + 0.6 determined by comparing array-based DSB signals with Southern blot-based DSB frequencies (Figure S7).
